# Impact of Adverse Metabolic Factors on the Burden of Chronic Kidney Disease in the Chinese Population ≥ 20 Years Old, 1990–2019

**DOI:** 10.1002/dmrr.70143

**Published:** 2026-02-27

**Authors:** Jiali Huang, Yiwei He, Fan Yang

**Affiliations:** ^1^ Department of Endocrine and Metabolism Geriatric Diseases Institute of Chengdu Chengdu Fifth People's Hospital (The Second Clinical Medical College, Affiliated Fifth People's Hospital of Chengdu University of Traditional Chinese Medicine) Chengdu China; ^2^ Department of Nephrology Xiangya Hospital Zhuzhou Central South University Zhuzhou China

**Keywords:** China, chronic kidney disease, disease burden, global burden of disease, metabolic risk factors

## Abstract

**Aims:**

To quantify temporal trends in chronic kidney disease (CKD) burden attributable to adverse metabolic risk factors among Chinese adults aged ≥ 20 years during 1990–2019.

**Materials and Methods:**

We used Global Burden of Disease Study 2019 (GBD 2019) estimates for China to assess five CKD causes (CKD due to type 1 diabetes, type 2 diabetes, hypertension, glomerulonephritis, and other/unspecified causes) and three metabolic risks (high systolic blood pressure (SBP), high body mass index (BMI), and high fasting plasma glucose (HFPG)). Outcomes were deaths and disability‐adjusted life years (DALYs) (numbers and rates per 100,000), stratified by sex and 13 age groups. Joinpoint regression was used to estimate annual percent change (APC) and average annual percent change (AAPC) in rates from 1990 to 2019.

**Results:**

In 2019, the mortality rate for CKD attributable to these metabolic risks was 6.87 per 100,000, and total attributable DALYs were 4201 (thousand). Hypertensive nephropathy had the largest attributable burden (mortality 2.5/100,000; DALY rate 59.2/100,000), followed by CKD due to type 2 diabetes. From 1990 to 2019, the overall attributable CKD mortality rate increased (AAPC = 0.55%) while the DALY rate decreased slightly (AAPC = −0.27%). High SBP remained the leading contributor to attributable burden in 2019, whereas high BMI showed the fastest increase in attributable mortality burden over time. Attributable burden increased steeply with age and was higher in males than in females.

**Conclusions:**

High SBP remains a major driver of CKD burden in China, but the rapidly rising high‐BMI‐attributable burden highlights the need for integrated prevention focused on blood pressure control and obesity prevention, particularly among older adults and men.

**Abbreviations**
AAPC: average annual percent change
APC: annual percent change
BMI: body mass index
CKD: chronic kidney disease
DALY: disability‐adjusted life year
GBD: Global Burden of Disease
HFPG: high fasting plasma glucose
IHME: Institute for Health Metrics and Evaluation
PAF: population‐attributable fraction
SBP: systolic blood pressure
TMREL: theoretical minimum risk exposure level
UI: uncertainty interval

## 1 Introduction

Chronic kidney disease (CKD) significantly increases morbidity and mortality from cardiovascular disease and other complications [1]. In 2023, the International Society of Nephrology Global Kidney Health Atlas (ISN‐GKHA) indicated that globally kidney disease is highly prevalent, costly to treat, and has a significant impact on health [2]. The current median global CKD prevalence is 9.5% and the median global CKD‐related mortality rate is 2.4% [2].

In recent years, the aetiology of CKD in patients in China has changed considerably, shifting from nephritis‐ and infection‐related nephropathies to predominantly metabolic disease‐mediated nephropathies. With socioeconomic development and lifestyle changes, adverse metabolic risk factors have increased, which are likely to affect CKD occurrence [3, 4].

Estimating the most recent prevalence of CKD helps to understand its disease burden and is an important tool to promote prevention and management. Some evidence suggests that CKD is increasingly detected at younger ages in China, underscoring the importance of early prevention and long‐term control of metabolic risk factors [5]. Additionally, the impact of adverse metabolic risk factors on the burden of CKD in China is not known.

This study used data from the Global Burden of Disease (GBD) released by the Institute for Health Metrics and Evaluation (IHME) to examine the evolving burden of CKD linked to adverse metabolic risk factors among Chinese individuals ≥ 20 years old from 1990 to 2019. The analysis aims to offer a scientific foundation for strategies to address metabolic risk factors early in order to prevent and treat CKD among people in China.

## 2 Materials and Methods

### 2.1 Data Source

The data for this study were obtained from GBD data published regularly by IHME. The most recent data were used, that is the GBD 2019 data (https://ghdx.healthdata.org/gbd‐2019), which includes data of more than 350 diseases and 199 risk factors in 195 countries worldwide from 1990 to 2019, and allows for the assessment of the disease burden of a wide range of diseases and risk factors globally [6, 7, 8, 9].

### 2.2 Extraction of Indicators and Definitions

Using the GBD 2019 Results Tool (https://vizhub.healthdata.org/gbd‐results/), we extracted estimates for China for calendar years 1990–2019, restricted to adults aged ≥ 20 years. We included five CKD causes as defined in GBD 2019: CKD due to type 1 diabetes, CKD due to type 2 diabetes, CKD due to hypertension, CKD due to glomerulonephritis, and CKD due to other and unspecified causes. We examined three adverse metabolic risk factors as defined in the GBD risk factor hierarchy: high systolic blood pressure (SBP), high body mass index (BMI), and high fasting plasma glucose (HFPG). In GBD, attributable burden reflects the comparative risk assessment framework, in which the population‐attributable fraction (PAF) is estimated relative to a theoretical minimum risk exposure level (TMREL) and applied to cause‐specific deaths and DALYs. Outcomes were deaths and DALYs (numbers and rates per 100,000), and we report 95% uncertainty intervals (UIs) when available. The study population was categorised into 13 age groups: 20–24, 25–29, 30–34, 35–39, 40–44, 45–49, 50–54, 55–59, 60–64, 65–69, 70–74, 75–79, and ≥ 80 years.

### 2.3 Statistical Methods

The primary indicators were mortality and disability‐adjusted life years (DALYs), including numbers and rates per 100,000. DALYs reflect years of life lost due to premature mortality plus years lived with disability. We used the Joinpoint Regression Programme (version 4.9.1.0) to analyse temporal trends in mortality and DALY rates. A log‐linear model was fitted to annual rates, allowing 0–4 joinpoints (given 30 annual observations). The final model was selected using the Monte Carlo permutation test (overall *α* = 0.05). We report annual percent change (APC) for each segment and average annual percent change (AAPC) for the overall 1990–2019 period with 95% confidence intervals. All analyses were stratified by sex and age group.

## 3 Results

### 3.1 Burden and Change in CKD Attributable to Adverse Metabolic Risk Factors in Chinese Persons ≥ 20 Years Old From 1990 to 2019

Data are summarised in Table 1. In 2019, the mortality rate of CKD attributable to adverse metabolic risk factors was 6.9 per 100,000 person, and total attributable DALYs were 4201 (thousand). The burden of hypertensive nephropathy attributable to adverse metabolic risk factors was the highest, with mortality and DALY rates of 2.5/100,000 and 59.2/100,000 persons, respectively. This was followed by type 2 diabetic nephropathy with a mortality and DALY rates of 2.22/100,000 and 57.53/100,000 persons, respectively. Compared with 1990, CKD mortality rates attributable to adverse metabolic risk factors increased by an average of 0.55% per year, with the largest increase occurring in hypertensive nephropathy (AAPC = 1.12%), followed by type 2 diabetic nephropathy (AAPC = 0.90%) and nephropathy from other causes (AAPC = 0.36%).

**TABLE 1:** Burden and temporal trends of chronic kidney disease (CKD) attributable to adverse metabolic risk factors among adults aged ≥ 20 years in China, 1990 and 2019.

CKD type	Mortality rate (per 100,000)			DALY rate (per 100,000)		
	1990	2019	AAPC (%)	1990	2019	AAPC (%)
Chronic kidney disease	5.83	6.88	0.56	219.84	201.26	−0.27
Chronic kidney disease due to type 1 diabetes	0.68	0.44	−1.48	30.39	17.14	−1.95
Chronic kidney disease due to type 2 diabetes	1.71	2.22	0.91*	52.6	57.53	0.38
Chronic kidney disease due to glomerulonephritis	0.64	0.6	−0.23	30.64	22.59	−1
Chronic kidney disease due to hypertension	1.79	2.47	1.13*	54.43	59.24	0.36*
Chronic kidney disease due to other and unspecified causes	1.02	1.14	0.37*	51.78	44.75	−0.45

*Note:* Rates are expressed per 100,000 population.

Abbreviations: AAPC, average annual percent change estimated using joinpoint regression; DALY, disability‐adjusted life year.

^*^ *p* < 0.05.

In contrast, type 1 diabetic nephropathy and nephropathy due to glomerulonephritis decreased by 1.48% and 0.23%, respectively. The DALY rate decreased by an average of 0.27% per year, with type 1 diabetic nephropathy showing the greatest decrease, followed by glomerulonephritis‐related nephropathy and other CKD not elsewhere classified (OCKDN); type 2 diabetic nephropathy exhibited the greatest increase.

### 3.2 Changes in Mortality and DALY Rates Attributable to Specific Adverse Metabolic Risk Factors, 1990–2019

**FIGURE 1:**
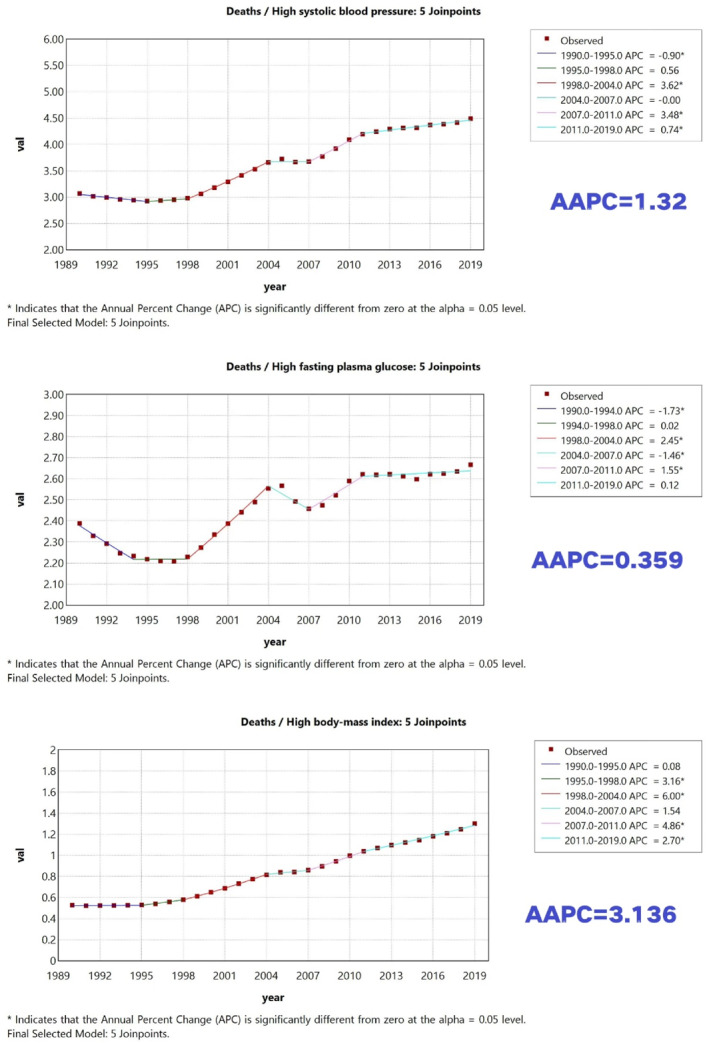
Trends in CKD mortality rates attributable to high SBP, high BMI, and HFPG in China (adults ≥ 20 years), 1990–2019. ‘Leading contributor’ refers to the highest absolute attributable rate in a given year, whereas ‘fastest increase’ refers to the largest AAPC over 1990–2019.

**FIGURE 2:**
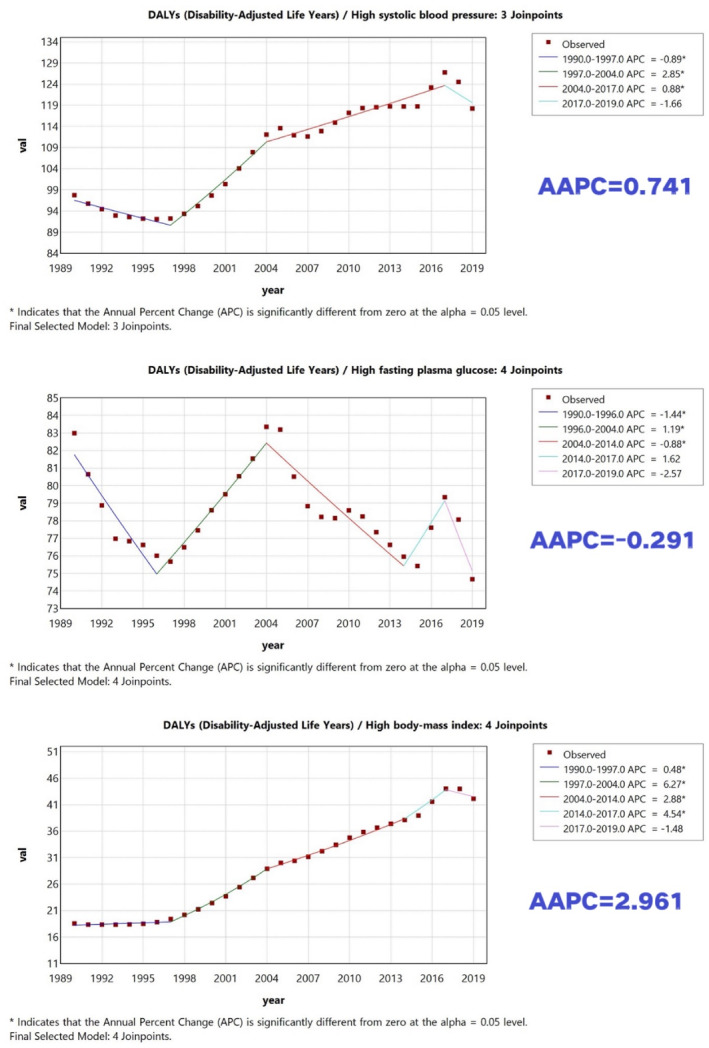
Trends in CKD DALY rates attributable to high SBP, high BMI, and HFPG in China (adults ≥ 20 years), 1990–2019. ‘Leading contributor’ refers to the highest absolute attributable rate in a given year, whereas ‘fastest increase’ refers to the largest AAPC over 1990–2019.

CKD mortality rates attributable to adverse metabolic risk factors (high BMI, HFPG, and high SBP) increased by varying magnitudes from 1990 to 2019 (Figures 1 and 2). High BMI showed the largest increase in attributable mortality rate over time (AAPC = 3.14%), followed by high SBP (AAPC = 1.32%) and HFPG (AAPC = 0.36%). Importantly, we distinguish growth over time from absolute contribution: high SBP remained the leading contributor to attributable mortality and DALY burden in 2019, whereas high BMI showed the fastest increase in mortality burden over 1990–2019. For DALYs, the overall attributable DALY rate decreased slightly over 1990–2019; HFPG was the only risk factor with a decreasing attributable DALY rate (AAPC = −0.29%), while high SBP and high BMI increased, with high BMI showing the largest increase in attributable DALY rate (AAPC = 2.96%).

### 3.3 Burden of CKD Attributable to Adverse Metabolic Risk Factors in Different Sexes and Age Groups

In general, the burden of CKD attributable to adverse metabolic risk factors increased with age and was higher in males than in females (Tables 2 and 3). Age gradients were steep: individuals aged ≥ 80 years had the highest attributable burden in 2019 (mortality rate 201.52/100,000; DALY rate 2517.42/100,000). In 2019, the 20–24 years old age group showed the most significant decrease in mortality and DALY rates compared with 1990, with reductions of 2.59% and 2.03%, respectively. On the other hand, the 60–79 years old age group had the smallest reductions in mortality and DALY rates, with decreases of 0.17% and 0.20%, respectively. Across all age groups, males experienced higher attributable mortality and DALY rates than females, consistent with reported sex differences in CKD outcomes.

**TABLE 2:** Age‐ and sex‐specific mortality rates for chronic kidney disease (CKD) attributable to adverse metabolic risk factors, China, 1990 and 2019.

Age group (years)	Total			Female			Male		
	1990	2019	AAPC (%)	1990	2019	AAPC (%)	1990	2019	AAPC (%)
20+ years old	5.83	6.88	0.56*	5.79	6.42	0.36*	5.8	7.34	0.83*
20–24 years	1.96	0.89	−2.59	1.46	0.69	−2.56	2.3	1.08	−2.48
25–29 years	2.48	1.11	−2.56	2.09	0.81	−3.22	2.73	1.4	−2.32
30–34 years	3.77	1.78	−2.45	3.47	1.14	−3.77	4.16	2.4	−1.85
35–39 years	5.7	2.64	−2.57	5.27	1.72	−3.79	6.14	3.53	−1.84
40–44 years	8.08	3.95	−2.48	7.31	2.78	−3.28	8.81	5.06	−1.94
45–49 years	9.72	5.12	−2.12	8.71	4.07	−2.59	10.07	6.12	−1.71
50–54 years	14.24	8.14	−1.93	14.34	6.82	−2.53	14.12	9.46	−1.35
55–59 years	19.14	12.38	−1.59	20.49	10.91	−2.15	18.23	13.84	−0.95
60–64 years	25.81	19.9	−0.91	27.53	17.71	−1.51	24.65	22.07	−0.38
65–69 years	36.7	33.15	−0.32	37.16	30.76	−0.65	36.43	35.64	−0.02
70–74 years	56.5	58.4	0.19*	51.42	52.93	0.1	62.14	64.14	0.18
75–79 years	94.23	96.86	0.11	88.66	87.13	−0.06	102.82	107.67	0.21
80+ years old	166.77	201.52	0.61*	150.06	173.41	0.5*	203.69	245.67	0.68*

*Note:* Rates are expressed per 100,000 population.

Abbreviation: AAPC, average annual percent change estimated using joinpoint regression.

^*^ *p* < 0.05.

**TABLE 3:** Age‐ and sex‐specific disability‐adjusted life year (DALY) rates for chronic kidney disease (CKD) attributable to adverse metabolic risk factors, China, 1990 and 2019.

Age group (years)	Total			Female			Male		
	1990	2019	AAPC (%)	1990	2019	AAPC (%)	1990	2019	AAPC (%)
20+ years old	219.84	201.26	−0.27	219.09	187.64	−0.49	220.57	215.09	−0.05
20–24 years	169.6	92.33	−2.02	162.78	88.04	−1.97	176.12	96.25	−1.97
25–29 years	206.92	116.34	−1.91	207.68	107.88	−1.96	206.21	124.5	−1.7
30–34 years	282.3	165.2	−1.61	274.04	139.57	−2.21	289.88	190.25	−1.31
35–39 years	374.44	217.08	−1.8	361.27	179.79	−2.29	386.74	253	−1.4
40–44 years	472.43	288.42	−1.63	438.64	243.91	−1.97	503.09	331.12	−1.29
45–49 years	512.56	339.21	−1.14	500.5	305.18	−1.38	523.36	371.96	−1.12
50–54 years	645.36	443.27	−1.23	658.27	401.2	−1.64	633.89	484.96	−0.86
55–59 years	765.71	572.82	−1.01	803.34	527.88	−1.28	731.54	617.35	−0.55
60–64 years	893.65	754.39	−0.53	931.97	697.01	−0.86	857.58	811.22	−0.16
65–69 years	1074.81	1006.86	−0.2	1089.83	961.48	−0.38	1059.29	1053.92	0.09
70–74 years	1369.76	1414.4	0.2	1291.13	1328.69	0.19	1460.7	1504.52	0.17
75–79 years	1804.43	1853.98	0.13	1711.31	1723.26	0.05	1927.69	1999.25	0.18
80+ years old	2207	2517.42	0.49	1966.08	2199.49	0.48	2640.65	3016.56	0.48

*Note:* Rates are expressed per 100,000 population.

Abbreviations: AAPC, average annual percent change estimated using joinpoint regression; DALY, disability‐adjusted life year.

* *p* < 0.05.

For CKD overall (adults ≥ 20 years), the mortality and DALY rates linked to adverse metabolic risk factors in 2019 were 7.34 per 100,000 and 215.09 per 100,000 in males, respectively, higher than those in females (6.42 per 100,000 and 187.64 per 100,000, respectively).

## 4 Discussion

Despite advances in scientific research that have led to a better understanding of disease pathogenesis and significant progress in disease management, the mortality associated with CKD has not improved significantly compared with other non‐communicable diseases [10]. CKD is defined by a gradual reduction in kidney function that ultimately results in end‐stage renal disease, which is typically irreversible.

Over the past 3 decades, age‐specific CKD mortality has increased markedly with age, with an exponential rise after approximately 60 years in China, highlighting the importance of prevention and management among older adults [11].

This pattern is consistent with population ageing and the higher prevalence of hypertension, diabetes, and obesity in older adults. To reduce CKD mortality, targeted health management and early intervention in middle‐aged and elderly populations are needed, including prevention, diagnosis, and treatment, alongside sustained lifestyle improvement.

In order to effectively reduce the number of deaths from CKD, the most cost‐effective strategy focuses on preventing its occurrence. This requires accurate identification and screening of high‐risk populations, as well as interventions targeting key risk factors. Among numerous risk factors, elevated SBP, increased HFPG, and high BMI are notable for their substantial impact on the development and progression of CKD.

High SBP remained the leading contributor to attributable burden in 2019, whereas high BMI increased the fastest over 1990–2019.

Age‐ and sex‐specific patterns are clinically and programmatically relevant. Prior work based on GBD 2019 has reported higher CKD prevalence in females but higher mortality in males, suggesting differences in progression, access to care, competing risks, and/or risk‐factor control [11]. A comprehensive review also notes that kidney function tends to decline faster in men and that mortality among individuals with predialysis CKD is often higher in men, whereas women may have different care pathways at older ages [12]. These findings support targeted screening and integrated management of blood pressure, glycaemia, and weight—particularly among older adults and men.

### 4.1 Potential Drivers in the Chinese Context

Several China‐specific factors may contribute to the observed trends. First, rapid population ageing increases the number of individuals at risk of CKD and amplifies the absolute burden [13]. Second, the nutrition transition has shifted dietary patterns towards higher fat and animal‐source foods and greater consumption of industrially processed foods, alongside reduced physical activity—are linked to rising overweight/obesity and cardiometabolic disease risk [14, 15, 16]. Third, national surveys show that hypertension prevalence increased substantially between 2004 and 2018, even as awareness and control improved, implying that the pool of individuals exposed to high SBP remains large [17]. Similarly, nationally representative data show an increasing prevalence of diabetes in China from 2013 to 2018, which may contribute to the rising burden of diabetic CKD [18]. Finally, health‐system policies—such as the Healthy China 2030 initiative and primary care‐based hypertension management programs—may improve detection and treatment over time, potentially affecting observed trends and age/sex differentials [19, 20].

### 4.2 Limitations

This study has limitations. As a secondary analysis of modelled GBD estimates, results depend on the quality of underlying data sources and modelling assumptions, and uncertainty may be larger for some subgroups. Risk‐attributable estimates reflect comparative risk assessment assumptions (including TMREL) rather than causal effects at the individual level. We analysed national‐level trends and cannot account for individual‐level confounding, regional heterogeneity, or clinical factors such as CKD stage, albuminuria, and treatment patterns. Future work combining GBD with high‐quality cohort and registry data may better clarify mechanisms and guide policy.

## Author Contributions

**Jiali Huang:** investigation, methodology, writing – original draft, writing – review and editing. **Yiwei He:** writing – review and editing. **Fan Yang:** formal analysis, writing – review and editing.

## Funding

The authors have nothing to report.

## Ethics Statement

The authors have nothing to report.

## Consent

The authors have nothing to report.

## Conflicts of Interest

The authors declare no conflicts of interest.

## Data Availability

GBD 2019 data are available from IHME (https://ghdx.healthdata.org/gbd‐2019) and the GBD Results Tool.
